# Group Well Child Care for Mothers with Opioid Use Disorder: Framework for Implementation

**DOI:** 10.1007/s10995-023-03762-w

**Published:** 2023-07-29

**Authors:** Neera Goyal, Meghan Gannon, Erica Sood, Grace Harris, Elizabeth Franko, Diane J. Abatemarco, Dennis J. Hand, Susan Leib, Vanessa L. Short

**Affiliations:** 1 Nemours Children’s Health, Wilmington, DE USA; 2 Sidney Kimmel Medical College of Medicine, Thomas Jefferson University, Philadelphia, PA USA; 3 Sidney Kimmel Medical College of Nursing, Philadelphia, PA USA; 4 Department of Pediatric and Adolescent Medicine, Einstein Medical Center Philadelphia, Philadelphia, PA USA; 5 Nemours Children’s Health Philadelphia, 833 Chestnut St, Ste. 300, Philadelphia, PA 19107 USA

**Keywords:** Opioid use disorder, Group well child care, Implementation, Pediatric, Perspectives

## Abstract

**Objective:**

For parents with opioid use disorder (OUD) and their children, group well child care (WCC) is an under-studied intervention that may reduce stigma, increase quality of care, and improve clinical outcomes. We explored barriers and facilitators to this intervention using an implementation science framework.

**Methods:**

A qualitative study was conducted from October 2020–March 2021 as part of the planning phase of a cluster-randomized trial of group WCC. Parent participants were recruited from one urban, university-affiliated OUD treatment center to participate in semi-structured telephone interviews. Eligible parents had a child under two years old and were English speaking. Clinician participants were recruited from a nearby pediatric primary care practice. Inductive thematic analysis of interview responses was led by two investigators using open coding procedures.

**Results:**

Thirty-one parents and thirteen pediatric clinicians participated in the interviews. Most parents (68%) reported that they would be likely or very likely to bring their child to the OUD treatment center for WCC. Six themes emerged describing perceived implementation barriers, including intervention difficulty, complexity, and potential negative outcomes such as loss of privacy. Six themes emerged as implementation facilitators: (1) focus on parental OUD and recovery, (2) peer support, (3) accessibility and coordination of care, (4) clinician skill and expertise in parental OUD, (5) increased time for patient care, and (6) continuity of care.

**Conclusions for Practice:**

Parents and clinicians expressed multiple perceived benefits of this intervention. Identified barriers and facilitators will inform implementation and evaluation of group WCC within one OUD treatment program.

**Supplementary Information:**

The online version contains supplementary material available at 10.1007/s10995-023-03762-w.

## Significance

*What is Already Known on the Subject?* Birthing parents with opioid use disorder and their children often face social and economic hardships, including barriers to well child care engagement. Group well child care has improved family outcomes in other patient populations.

*What this Study Adds?* Birthing parents in treatment for opioid use disorder and pediatric clinicians identify potential facilitators of implementing group well child care within a parental treatment program; these facilitators include a focus on parental recovery and wellness, peer support, and accessibility and coordination of care.

**Supplementary Information:**

The online version contains supplementary material available at 10.1007/s10995-023-03762-w.

## Introduction

Opioid use disorder (OUD) during pregnancy has emerged as a major United States (US) public health issue, with considerable focus on perinatal care of affected parents and newborns (Grossman et al., 2018; Kaplan et al., 2020; Patrick, 2017). Fewer studies have focused on pediatric primary care for these families, where risks for child health and development including poverty, stigma, and social isolation require ongoing attention (Gressler et al., 2019; Pryor et al., 2017; Sutter et al., 2017). Well child care (WCC) represents a critical opportunity for family support (American Academy of Pediatrics, 2023). Unfortunately, available studies suggest that despite multiple clinical concerns in infancy and early childhood among these families, attendance to recommended WCC visits is low (Beldick et al., 2023; Goyal et al., 2020; Jarlenski et al., 2020). Parents with OUD have reported their WCC experiences as lacking key aspects of family centeredness such as open communication and personalized care (Short et al., 2019, 2022). Simultaneously, pediatricians report a lack of time and resources to comprehensively address their questions and psychosocial needs (Rohde et al., 2023).

Group WCC, in which a group of families with similarly aged children receive WCC together during a longer visit, is one intervention which may engage, support, and empower parents with OUD. With group WCC, regularly scheduled WCC visits focus on child health and development, but also incorporate group discussion of topics such as family planning and stress reduction. Session planning guides are available through Centering Healthcare Institute that align with American Academy of Pediatrics recommendations (Centering Healthcare Institute [CHI], 2023). In addition to routine anticipatory guidance, these session planning guides include structured parental mindfulness exercises to reduce stress and promote emotional awareness for parents, as well as hands-on activities such as infant play. Thus, group WCC has unique potential to promote information sharing and peer support across multiple domains of parental and child wellness (Connor et al., 2018; DeLago et al., 2018; Platt et al., 2022). Prior studies suggest enhanced family resilience and interconnectedness, improved visit attendance, and increased satisfaction with care (Fenick et al., 2020; Irigoyen et al., 2021; Oldfield et al., 2020). Despite these benefits, group WCC remains underutilized, with numerous logistical challenges that include cost, space, and scheduling (Gaskin et al., 2021; Gresh et al., 2023).

To date, research on group WCC for parents in treatment for OUD is limited. For this population, prior studies suggest an increased risk of maladaptive parenting, including heightened tendency towards harsh punishment, low responsiveness to child cues, and knowledge gaps regarding child development (Maguire et al., 2016; Rizzo et al., 2014; Sutter et al., 2017). Addressing these risks during WCC visits is a considerable challenge for pediatricians (Abatemarco et al., 2008, 2018). For these families, group WCC may improve child outcomes through multiple mechanisms, including healthcare engagement, parenting knowledge, and parent–child attachment. For parents with OUD, who often have significant trauma burden, parenting stress, and depressive symptoms, mindfulness may be a particularly useful tool within group WCC to strengthen their capacity to respond, interact, and attune to their child (Gannon et al., 2017).

For the current study, we utilized an implementation science framework to explore factors that may impact the adaptation and delivery of group WCC for parents with OUD. Implementation science is increasingly recognized as the exploration of factors that may impede or support the real-world success of a given intervention, ultimately affecting efficacy, sustainability, and scalability (Damschroder et al., 2009). Given the complexity of group WCC as an intervention, and the clinical and psychosocial challenges of caring for parents with OUD and their children, we sought to understand barriers and facilitators to implementation.

## Methods

This qualitative study was conducted in the planning phase of a cluster-randomized trial of group WCC co-located within an urban, university-affiliated OUD treatment program in Pennsylvania that provides medications for OUD (MOUD), therapy, case management and supportive services for pregnant and parenting women with OUD. Women eligible for this study were (1) receiving OUD services in the treatment program, (2) parent to a child under two years old, and (3) English-speaking. Participants also included clinicians (attendings, resident physicians, and nurses) from an urban, university-affiliated pediatric primary care practice predominately serving Medicaid-insured patients.

### Procedures and Data Sources

Study procedures were conducted by our research team comprised of a public health researchers, epidemiologists, and psychologists. All procedures were conducted in accord with prevailing ethical principles and were reviewed and approved by relevant Institutional Review Boards. A waiver of written informed consent was granted, however all study participants provided verbal informed consent. Parental recruitment was conducted using convenience sampling to identify potential participants. Parents were telephoned to determine eligibility and ascertain interest in participation. A target sample size of 30 parents was planned to achieve thematic saturation (Demianczyk et al., 2022; Hennink & Kaiser, 2022). Enrolled parents were scheduled for a one-time, 30 min, semi-structured telephone interview. All interviews were conducted between October 2020 and January 2021 by one of two study team members. Each interview began with a brief survey asking participants to report demographics and information about their child’s healthcare experience. Parents were compensated for their time with $50.

All clinicians at the pediatric practice were recruited with an email containing a brief study description and contact information, and a reminder email one month later. We set an initial target sample size of approximately 15 clinicians, with plans to collect additional data if thematic saturation was not achieved (Hennink & Kaiser, 2022). Clinicians were interviewed by telephone between January and March 2021 by one study team member. Interviews were 30 min and semi-structured, and began with a brief survey asking about demographics and OUD training or education. No monetary compensation was provided.

Interview guides were developed using the Consolidated Framework for Implementation Research (CFIR) Guidelines (Damschroder et al., 2009). This framework contains multiple domains that can guide the assessment of potential barriers and facilitators to effective implementation (see Online Resource 1). As our study focused on pregnant and parenting women in treatment for OUD and their relationship as a mother to their child, the terms ‘mother’ and ‘maternal’ were used during the interviews, and for consistency will be used for the remainder of this report (National Institutes of Health, 2023). Because all interviews were conducted prior to group WCC implementation, the interview included the following brief description of group WCC to orient study participants: “With group pediatric care, a group of five to six moms and their infants see a pediatrician together for regular checkups. Each visit would last about an hour and a half, where everyone had some one-on-one time with the doctor, followed by time for group discussion about different topics, as well as some time to focus on mom’s health and wellness too. Each child would receive regular vaccines and screenings as part of the visit.” The interview continued with discussion prompts to explore barriers and facilitators to group WCC. Participants were specifically prompted to provide their perspective on mindfulness, which is a unique aspect of group WCC compared with routine WCC (CHI,  2023). For example, clinicians were asked, “Would you feel comfortable leading short breathing exercises or talking generally about mindfulness?” All interviews were audio recorded and transcribed with removal of identifiers. Interview transcripts were uploaded into Dedoose V 8.0.35 for qualitative coding and analysis.

### Data Analyses

Survey data were analyzed using descriptive statistics. Interview data were analyzed using an inductive thematic approach. Thematic analysis focuses on the subjective human experience and emphasizes participants’ experiences, feelings and perceptions (Braun & Clarke, 2006). Coding was led by two researchers with support from three medical students. Coders first independently reviewed seven transcripts with preliminary thematic categories in mind to develop maternal and clinician codebooks. Discrepancies between coders were examined and discussed, and the codebooks were refined. Another nine transcripts were then coded by the two primary coders to establish inter-coder reliability (pooled Cohen’s Kappa coefficient; maternal K = 0.87 and clinician K = 0.85), after which the remaining transcripts were divided among the two primary coders.

Transcript excerpts were organized into emergent themes. Because our interviews included specific questions about mindfulness in group WCC as a potential benefit, participant responses regarding barriers and facilitators to mindfulness activities were analyzed separately to avoid biasing results. Thematic saturation was reached when no new themes were identified, separately for maternal and clinician data (Starks & Trinidad, 2007). Findings were shared with two maternal and two clinician participants to confirm or challenge findings. Debrief sessions were also held with other research team members to further validate or challenge interpretations of the data. Results are reported in accordance with Consolidated Criteria for Reporting Qualitative Research Guidelines (Tong et al., 2007).

## Results

### Participant Characteristics

Of 76 mothers contacted for recruitment, 31 enrolled and completed telephone interviews. The majority (84%) endorsed having received some type of therapy or service in a group format, and of those participants, 73% rated their overall experience with receiving care in a group format as either excellent or very good. Most (68%) reported that they would be likely or very likely to bring their child to the OUD treatment center for WCC. Of 53 clinicians invited to participate, 13 enrolled and completed telephone interviews. All clinician participants reported experience working with mothers receiving MOUD, and most (92%) had received some form of training about substance use disorders during their careers (see Table 1).

**Table 1:** Questionnaire responses of participants

Maternal participants (N = 30)	
Age in years, median (standard deviation)	33 (5.4)
Ethnicity n (%)	
Hispanic	3 (10)
Non-hispanic	28 (90)
Race n (%)	
White	25 (83)
Black or African-American	3 (10)
Other	2 (7)
Marital status n (%)	
Single	23 (77)
Married	3 (10)
Divorced/separated or widowed	4 (13)
Education level n (%)	
Some high school	8 (27)
High school graduate or GED	7 (23)
Some college education or 4-year college graduate	16 (53)
Child received pharmacotherapy for opioid withdrawal n (%)	
Yes	20 (67)
No	11 (33)
Number of children taken care of at home, median (standard deviation)	1 (1.0)
Age of youngest child in months, mean (range)	14 (1–24)

Clinician participants (N = 13)	
Age in years, median (standard deviation)	32.5 (5.4)
Sex	
Female	12 (92)
Male	1 (8)
Ethnicity	
Hispanic	1 (8)
Non-hispanic	12 (92)
Race	
White	12 (92)
Black or African-American	1 (8)
Role as provider	
Attending physician	9 (69)
Resident physician	3 (23)
Nurse	1 (8)
Education, training, and confidence with opioid use disorder	
Prior education or training in opioid use disorder	12 (92)
History working with children of mothers with opioid use disorder	13 (100)
Reported feeling very confident about working with families affected by maternal opioid use disorder	7 (54)

Six themes emerged corresponding with potential barriers to implementing group WCC (Table 2), and six themes emerged as potential facilitators (Table 3). Thematic saturation was achieved by the seventeenth interview for mothers and the fourth interview for clinicians.

**Table 2:** Barrier themes and representative quotes

	Theme	Representative quote
Barrier	Intervention complexity: Resource intensiveness *6% of Mothers* *85% of Clinicians*	• “I personally would not want to be taking a lot of notes because I'd want to dedicate the visit to really addressing the family's concerns and being with the patient. I think then, documenting after the fact and remembering the different interactions with each different family and provider, our patient and parent, and doing thorough documentation would be difficult.” (Clinician) • “I think that space is always limited, so it would be needing a room and then needing little pop-off areas to do the individual care. So, sort of, coordinating that. I think we would need to have the appropriate ancillary staff. Say they needed to have shots. We would need to have a medical assistant, and it would be nice if the case manager and social worker could be there. So just organizing the players that would need to do this.” (Clinician)
	Loss of privacy as a negative outcome *42% of Mothers* *31% of Clinicians*	• “Her special things like her club foot. I don’t know how open I am about that, because not everyone has knowledge about it. So I don’t know if I would talk about that there.” (Mother) • “Downsides, I think would be if moms are hesitant to speak up because they are in a group setting or if there was something more confidential, personal. I don’t know if there’s time built into the group well visit to have one-on-one time with each parent, or is the entire visit conducted as a group. I think if there are more private or personal issues that need to be addressed, I don’t know exactly how that would go…” (Clinician)
	Intervention difficulty: addressing comprehensive medical and psychosocial care needs *6% of Mothers* *69% of Clinicians*	• “It would just feel almost like a class, not an appointment. So I like to be able to ask questions or just anything, I wouldn’t be able to ask because there’s so many other moms and kids and they have a lot at one time, so maybe it wouldn’t feel like all your needs can be met.” (Mother) • “I think that it would work best for a healthy population, that if there’s a child who has specific medical needs, those I again, would worry, it would be tough to devote enough time to that in a group model.” (Clinician)
	Intervention complexity: Intricacies of scheduling *19% of Mothers* *46% of Clinicians*	• “Say it’s my baby’s appointment, but I had my daughter, would she be allowed to come too? Or would she be a distraction? That’s other stuff that I would consider, if they’re going to have childcare, or with other kids what I would do during that setting if that applied.” (Mother) • “Again, it’s mostly logistics. So like having the appointment slot be at a time that would work best both for the patients and for my own schedule. So sometimes carving out time for an appointment takes more time… if it was first thing in the morning, fine, that's relatively easy. But if it was some point carved into the day, making sure that I'm done with the previous patients before it, getting to the place that it was at, those types of challenges for sure” (Clinician)
	Intervention difficulty: Managing group dynamics *19% of Mothers* *69% of Clinicians*	• “Everyone's at different stages when you come to the [treatment center] and that’s not necessarily a bad thing, but it can be a difficult thing.” (Mother) • “I think you can sometimes have people that dominate the small groups. You have to learn how to make it a safe space for everyone to be able to participate, so have sort of rules of engagement established.” (Clinician)
	Increased exposure to COVID-19 as a negative outcome *87% of Mothers* *92% of Clinicians*	• “Just I really think everybody should wear a mask and keep their distance and nobody should be touching other people’s children. Because both my kids got chronic asthma and me and my husband are both HIV positive, so we’re very at-risk. If we get it we’ll get really sick.” (Mother) • “I don’t know how feasible it is to have all the children in one room together, which then obviously creates a little bit of an issue. But parent-wise, masks, making sure they're all sitting a decent amount apart, and then keeping the groups small enough that we can keep social distancing.” (Clinician)

**Table 3:** Facilitator themes and representative quotes

	Theme	Representative quote
Facilitators	Focus on maternal recovery and wellness as an intervention benefit *100% of Mothers* *100% of Clinicians*	• “If they’re new moms and they never had babies before, I think just education as far as methadone, how it… Especially if they’re new moms on methadone, if they’ve never been on methadone before, if they've never had a child on methadone before, and educating them on how this might work or how the babies might deal with coming into the world and what it might look like for them and what their care might look like and what the mother's care might look like and how they can be supported. Because that's really hard” (Mother) • “I think that it may be worth thinking about the patient education handouts that are given at the end, and whether it may be worth editing for some specific needs that this population may have.” (Clinician)
	Peer support as an intervention benefit *97% of Mothers* *85% of Clinicians*	• “There would probably be less judgment and at least two, like I would probably feel more comfortable because I'd already be in like an atmosphere where the other mothers are on the same note as me. And I'm also just like, again with being able to relate to other mothers, like they’re going through the same things that I am and their children most likely are too. Maybe they’re even going through things that my child might go through later that we’re getting like a head's up. So stuff like that.” (Mother) • “I think the reassurance of hearing other moms discuss the questions they have and what they're going through with their own babies would make a lot of moms feel like they're not alone and this is what everybody's going through and that would be reassuring. So I think that was definitely kind of the biggest benefit in terms of the care, and also that feeling of community, like you belong to this group, which I think would be really helpful for them.” (Clinician)
	Accessibility and coordination of care as an intervention benefit *87% of Mothers* *69% of Clinicians*	• “I think a lot more women are going to make sure that they show up to appointments because it's at the clinic too. It makes it a little bit more … one of the reasons I would do it is because I already go there and it would make a lot more sense to just make everything at one stop, like a one-stop shop, instead of traveling all over downtown.” (Mother) • “I think the biggest benefit is the one-stop shopping, because I think if you can have well-care provided for the child, get them vaccinated, address their issues, et cetera, and then have group counseling available… I think for mothers that are OUD, they have group and meetings to make and things like that. And so, I think if you could do it all at one time, I think you have a much better chance of them getting it more often and getting it on time.” (Clinician)
	Clinician skills and experience in caring for mothers with OUD *42% of Mothers* *85% of Clinicians*	• “I think it would be so useful, because just feeling comfortable around your own kind almost, and all that, and not feeling judged, and knowing that they have extra experience with people who suffer [from SUD].” [Mother] • “I feel like I don’t always address it head on with the family because I find it’s an awkward conversation to have and I don’t want to offend anyone by bringing it up. So I think it’s just an uncomfortable topic to talk about. So kind of more training on how to approach those conversations would be helpful.” (Clinician)
	More time for patient care as an intervention benefit *45% of Mothers* *38% of Clinicians*	• “Like I said, the most thing that I like is that they take their time…. They actually listen to what the problem is and they try to help you.” (Mother) • “I mean, if it works well, it can allow you to see more people and be a more effective educator and, ultimately, provide better care. It would be more streamlined instead of piecemeal. You could get to know the patients and their parents a lot better as well, and identify different risks or gaps in care.” (Clinician)
	Clinician continuity as an intervention benefit *16% of Mothers* *38% of Clinicians*	• “I think you’d have a better personal relationship with your doctor. I think that’s important because my daughter had a bunch of different pediatricians, it wasn’t the same doctor. If the group settings were like that I think that would help too, if it was the same primary one or at least if it was two practitioners… I feel like it's just more comfortable and maybe easier to talk to the doctor if you have a more familiar one time to time than just a rotating staff.” (Mother) • “I think it would definitely help if you were the same provider, the same doctor seeing these babies at every visit, then I think it would be nice to have a little bit better continuity of care then we get some time with these families.” (Clinician)

### Barriers

#### Intervention Complexity: Resource Intensiveness

Clinicians noted the need for equipment, supplies, and staff for routine WCC screenings and procedures during group WCC. They also noted the need for a large enough space with an adjacent private area for individual care. Some clinicians questioned whether it was possible to fit group content and individual time for each family into a 90 min session. Clinical documentation and limited reimbursement within a fee-for-service model were also raised as potential barriers.

#### Loss of Privacy as a Potential Negative Outcome

While some mothers noted that they would feel comfortable discussing most topics in a group, others stated discomfort discussing topics they perceived to be private or sensitive, such as their substance use history, their relationship with their partner/child’s father, or their child’s special healthcare needs. In some cases, mothers reported concern that other mothers in the group may judge their child or their ability as a parent. Several clinicians similarly expressed concern that mothers may be hesitant to speak about personal issues within the group setting and stated that one-to-one time with each mother to address these issues may be necessary.

#### Intervention Difficulty: Addressing Comprehensive Medical and Psychosocial Care Needs

Some clinicians questioned the appropriateness of addressing specific medical and developmental concerns within the group setting and how this attention towards an individual family could be balanced with attention towards the whole group. They also raised concerns about obtaining child/family history, assessing development, providing tailored education and recommendations, and answering specific questions that may relate to only one child in the group. When mothers expressed perceived challenges regarding attention to individual healthcare needs during group sessions, their concerns related primarily to their own privacy and comfort.

#### Intervention Complexity: Intricacy of Scheduling

Participants described scheduling challenges and competing demands as potential barriers to group WCC. Mothers identified job responsibilities and OUD treatment schedules as potentially conflicting across group participants. Clinicians noted the potential difficulty of fitting group sessions into their clinic schedule, emphasizing the need for dedicated time for group WCC rather than adding it to an already fully scheduled clinic day. Participants identified inclement weather and other unforeseen circumstances as additional challenges.

#### Intervention Difficulty: Managing Group Dynamics

Both mothers and clinicians discussed the potential challenge of including mothers in treatment for OUD at different stages of recovery and with different levels of knowledge regarding child development and parenting. Several mothers expressed concern for group disruptions from participants arriving late, not taking the group seriously, or consistently deviating from the discussion topic. Several clinicians expressed concern that mothers may bring outside interpersonal conflict into the group setting, that a small number of participants could dominate the discussion, or that comparisons between mothers or children could disrupt the group dynamic. The importance of ground rules developed in collaboration with group participants was emphasized by some clinicians. A few clinicians also noted concern that mothers may have difficulty focusing on the content in a noisy group setting or that clinicians may be pulled in different directions when trying to address maternal information needs.

#### COVID-19 Exposure as a Potential Negative Outcome

Participants noted that the COVID-19 pandemic could impede implementation, and that precautions such as masking and social distancing would mitigate risk. Several clinicians noted that mandatory vaccination would help but may not be feasible. Some mothers stated that a virtual format would be preferable for group WCC during the pandemic and that they would not likely participate if this option was unavailable. Several mothers emphasized the need to protect children and mothers with chronic health conditions that may place them at higher risk for COVID-19 complications.

### Facilitators

#### Focus on Maternal OUD and Recovery as an Intervention Benefit

Mothers described how content related to intergenerational substance use, effects of maternal substance use on child development, and maternal health/stress would be highly beneficial to include in group WCC. Both clinicians and mothers shared perspectives on potential for group WCC to facilitate health literacy through discussion and education around tailored health topics specific to children affected by maternal OUD.

#### Peer Support as an Intervention Benefit

Mothers and clinicians both spoke about how group WCC would normalize parenting challenges and reassure mothers. Mothers noted how group WCC would offer them an opportunity to build relationships with other mothers and allow their children to socialize with other children of their same age. They were supportive of the inclusion of other support persons in the group visit (i.e., partners, spouses, parents). Clinicians felt that community sharing would enrich the content they typically offer and provide more insight for them into this population, thereby enhancing the therapeutic relationship.

#### Accessibility and Coordination of Care as an Intervention Benefit

Overall, the co-location of pediatric care with maternal OUD treatment was viewed by participants as helpful to implementation. Most mothers remarked how co-location would make them feel more comfortable and would reduce barriers to access. Mothers were also interested in whether attendance to group WCC sessions could count towards their OUD treatment participation. Most clinicians remarked how they perceived it would create an enjoyable environment, both for patients and themselves.

#### Clinician Skills and Experience in Caring for Mothers with OUD

Participants spoke about how clinician training in substance use and experience working with families affected by maternal substance use would facilitate implementation, fostering a trusting and comfortable environment for pediatric care, and reducing stigma (Table 3). Additionally, clinicians noted training in group WCC facilitation would be imperative.

#### More Time for Patient Care as an Intervention Benefit

Clinicians and mothers perceived the longer visit duration in group WCC to support more time for discussion. Clinicians appreciated the opportunity to provide anticipatory guidance to multiple families in one session. They also described the longer visit as an opportunity to learn more about their patients, through more meaningful interactions and shared learning through discussion.

#### Clinician Continuity as an Intervention Benefit

Mothers discussed the value of continuity of care to facilitate trust and ease with the clinician. Clinicians discussed how group WCC would better facilitate the development of the therapeutic relationship through longer regular visits. They noted how this consistent interaction would nurture understanding and familiarity, leading to identification of care gaps or emergent health issues more readily.

### Incorporating Mindfulness

Emergent themes corresponding with potential barriers and facilitators to incorporating mindfulness into group WCC are reported in Table 4. Mothers and clinicians expressed interest and support for mindfulness activities as part of the intervention.

**Table 4:** Barriers and facilitators to incorporating mindfulness into group well child care

	Representative quotes
Barrier themes	
Difficulty concentrating with children present	• “While it’s during the visit, I think the useful part of that is that you are using your time effectively. The problem with it is, are we doing it with the baby right there? It’s really hard to focus. When I had to do it a couple times, I didn’t have anybody to watch my son. Thankfully, he was a good calm baby, but he had his moments. And it’s hard to go back into that focused relaxation once you’re out of it.” (Mother) • “I’m picturing as you’re talking about this is just five kids running around a room and just as if they’re on a playground and kind of going crazy. So I personally would find it hard to have some time to do mindfulness in that environment.” (Clinician)
Lack of clinician expertise leading mindfulness in group setting	• “I'm not sure I have enough experience or enough thought about if it would be specifically more beneficial or not to this population, but from the information that I know, especially with PTSD, patients, they do great with mindfulness and really do better.” (Clinician)
Longer visit duration a potential negative outcome	• “Yeah. I wouldn't want it like super long at all.” (Mother)
Facilitator themes	
Benefit of creating sense of presence and focus in the group	• “Once we’re breathing I think it creates a better atmosphere for us to listen and to pay attention and just overall do better in the group. I think it'd be beneficial.” (Mother) • “Yeah, I definitely think it would be helpful if we could have them do it. Absolutely. Because like I said, I feel like sometimes with their treatment or their medications their thoughts are a little bit disorganized and this might help them focus a little bit better on what’s coming.” (Clinician)
Benefit of reducing maternal stress/anxiety	• “I feel like it’s useful when you’re very overwhelmed or you have anxiety or you’re at your wits end with your kids some days. But I feel like in every day … yeah, I guess yeah, because it's not just about taking breaks and stuff, it's about being mindful.” (Mother) • “Oh, so helpful. Again, I think that the root of OUD often lies in self-medication for anxiety or depression and so that can often be a pre-existing concern for these families.” (Clinician)
Benefit of supporting parenting behaviors	• “Like when she’s doing her little screaming bouts and you want to rip your hair out and you’re just like, “Whew”. You have to walk away for a second. So yeah, maybe to learn some techniques to better deal with them situations and all that could be helpful.” (Mother) • “I think a lot of parents can get, especially in young children, can get very overwhelmed, and having the tools just know how to take a deep breath and be able to find some inner peace and stillness, I think would be really great. I think a bunch of parents are like, the kids are screaming all the time and I can find a break to just breathe and think to myself. I think having some strategies and some exercises would be helpful.” (Clinician)
Benefit of supporting maternal recovery efforts	• “I think more and more evidence is showing that we can all benefit from mindfulness. We may benefit from doing a minute or two of mindfulness with all of our patients, regardless of what their mental health concerns are, and I think a lot of treatment of opioid use disorder focuses on medication management and also modification of activity and avoiding triggers, but I think that mindfulness could also be a strategy for addressing cravings and that kind of stuff too, if that’s not already done.” (Clinician)
Benefit for child to learn mindfulness tools	• “I would recommend mindfulness overall, I think it’s amazing. I like my child to meditate and see it working.” (Mother) • “Whether it’s chaos in their personal life or the behavior of multiple children or that type of thing. So I think that it can certainly help with anxiety and a general sense of lack of calm and that we know that, that affects baby too. If mom feels tense or uptight or anxious that, that is going to affect baby’s behavior and that type of thing. So I think it would be a positive benefit to both the moms and the kids.” (Clinician)

## Discussion

In this qualitative study, attitudes towards group WCC among mothers with OUD and clinicians seem to support our hypotheses that this intervention will improve engagement in care, parenting knowledge, and maternal-child attachment. However, several challenges may impede successful implementation if not appropriately addressed.

The potential barriers raised by our participants align with previously reported financial, structural, and procedural factors that have been shown to be important for group WCC implementation (Gresh et al., 2023). As participants have noted, staffing, training, space, and equipment are required for group WCC, underscoring the importance of a supportive policy environment and commensurate funding streams (Gresh et al., 2023; Connor et al., 2018). Examples of potential financial support include the federally-funded Healthy Start initiative, state-level funds, and in-kind service grants from Centering Healthcare Institute (National Healthy Start Association, 2023; CHI, 2023). For our project, upfront costs for staffing and equipment are supported through a federal research grant as a randomized clinical trial (Short et al., 2023). Long term, the cost effectiveness of group WCC relates to several factors including patient volume and clinician productivity (Yoshida et al., 2014). Although group WCC is typically reimbursed under traditional fee-for-service payment models, there are promising examples across the U.S. of enhanced reimbursement for group visits under a value-based payment model (Prenatal-to-3 Policy Impact Center, 2023; CHI, 2019). To maintain a sufficient volume of mothers with OUD, we anticipate needing flexible enrollment procedures, and frequent communication with high-risk obstetrical providers, birth hospitals, and OUD treatment staff. Scheduling for group WCC will require coordination between OUD treatment staff and pediatric clinical staff in the form of regular, multidisciplinary meetings.

Although most mothers in our sample reported positive experiences with group formatted care, concerns about privacy and group dynamics were raised by study participants. As recommended for all group WCC programs, we plan to use a large room with enough physical space to accommodate group activities as well as semi-private discussions (Gresh et al., 2023). Interdisciplinary co-facilitation is also generally recommended to assist the primary clinician in navigating group dynamics (Griswold & Walker, 2021; Marchel et al., 2015). Given that our group WCC sessions may include mothers in different stages of OUD recovery, we plan to incorporate a nurse practitioner from the OUD treatment staff as a co-facilitator with content expertise in OUD and familiarity with the treatment circumstances for each mother.

In order to ensure that group WCC meets the comprehensive care needs of each family, we will develop a health assessment questionnaire for group visits to facilitate history taking and identify specific concerns, as is common in other group WCC programs (Gresh et al., 2023). We will also tailor the content of group discussions to reflect parental priorities for anticipatory guidance (Goyal et al., 2023). Furthermore, we will develop printed informational handouts about these topics that are visually pleasing and easy to read. We will conduct semi-structured, individual interviews with mothers receiving group WCC during implementation to obtain feedback on group visit content, formatting, and written materials (Short et al., 2023).

COVID-19 emerged as a potential barrier, although this topic was specifically prompted. Of note, we conducted this study in late 2020, before COVID-19 vaccination became widely available. Given the continually evolving public health response to COVID-19, we anticipate that clinician and maternal attitudes will also change. Currently, our findings support the use of available federal guidance towards risk reduction, i.e. masking and encouraging vaccination (Centers for Disease Control and Prevention, 2023).

Finally, participants were overall supportive of mindfulness activities but identified potential barriers to this aspect of group WCC. Multiple clinician resources for integrating mindfulness into group WCC sessions in the form of brief breathing or movement exercises are available through Centering Healthcare Institute. We anticipate that this particular aspect of group WCC will align well with the trauma-informed, mindfulness-based practices already being implemented at this OUD treatment center (Gannon et al., 2017).

### Limitations

Because participants reported their projected and not actual experiences with group WCC, these data should be interpreted with caution. Furthermore, social desirability and selection bias may have impacted responses, particularly given the response rate < 50% for both maternal and clinician participants. Clinicians who participated were mostly non-Hispanic White and female, and purposive recruitment was not used to increase sample diversity. The maternal participant sample was also disproportionately non-Hispanic White compared with our local maternal OUD treatment population, which is estimated to be approximately two-thirds non-Hispanic White and at least 20% non-Hispanic Black. Given known racial-ethnic disparities in access to MOUD, future research should focus on the perspectives of minoritized parents with OUD and interventions to improve health equity (Gao et al., 2022; Tucker, 2022).

## Conclusions

Group WCC is perceived to confer multiple advantages over traditional WCC for mothers with OUD, but with multiple implementation challenges. Findings are being used to adapt group WCC for mothers with OUD and their children, and to integrate this intervention within one maternal OUD treatment program. Future research will study implementation outcomes such as attendance and fidelity to the intervention, as well as family outcomes (WCC engagement and experience, parenting knowledge, and maternal-child attachment).

### Supplementary Information

Below is the link to the electronic supplementary material.

Supplementary file1 (PDF 126 kb)

## Data Availability

Data is available from the authors upon request.

## Abbreviations

AAP: American academy of pediatrics
CHI: Centering healthcare institute
MOUD: Medications for opioid use disorder
OUD: Opioid use disorder
WCC: Well child care
