# Correction to: Group Well Child Care for Mothers with Opioid Use Disorder: Framework for Implementation

**DOI:** 10.1007/s10995-023-03774-6

**Published:** 2023-09-28

**Authors:** Neera Goyal, Meghan Gannon, Erica Sood, Grace Harris, Elizabeth Franko, Diane J. Abatemarco, Dennis J. Hand, Susan Leib, Vanessa L. Short

**Affiliations:** 1 Nemours Children’s Health, Wilmington, DE USA; 2 Sidney Kimmel Medical College of Medicine, Thomas Jeferson University, Philadelphia, PA USA; 3 Sidney Kimmel Medical College of Nursing, Philadelphia, PA USA; 4 Department of Pediatric and Adolescent Medicine, Einstein Medical Center Philadelphia, Philadelphia, PA USA; 5 Nemours Children’s Health Philadelphia, 833 Chestnut St, Ste. 300, 19107 Philadelphia, PA USA

**Correction to: Maternal and Child Health Journal**

10.1007/s10995-023-03762-w

The article ‘‘Group Well Child Care for Mothers with Opioid Use Disorder: Framework for Implementation’’, written by Neera Goyal, Meghan Gannon, Erica Sood, Grace Harris, Elizabeth Franko, Diane J. Abatemarco, Dennis J. Hand, Susan Leib and Vanessa L. Short was originally published electronically on the publisher’s internet portal on 29 July 2023 without open access. With the author(s) decision to opt for Open Choice, the copyright of the article changed on 24 August 2023 to The Author(s) 2023 and the article is forthwith distributed under a Creative Commons Attribution 4.0 International License, which permits use, sharing, adaptation, distribution and reproduction in any medium or format, as long as you give appropriate credit to the original author(s) and the source, provide a link to the Creative Commons licence and indicate if changes were made. The images or other third-party materials in this article are included in the articles Creative Commons licence, unless indicated otherwise in a credit line to the material. If material is not included in the articles Creative Commons licence and your intended use is not permitted by statutory regulation or exceeds the permitted use, you will need to obtain permission directly from the copyright holder. To view a copy of this licence, visit http://creativecommons.org/licenses/by/4.0.

