# Supplementary material for: Group Well Child Care for Mothers with Opioid Use Disorder: Framework for Implementation
Source: Matern Child Health J. 2023 Jul 29;27(Suppl 1):75–86. doi: 10.1007/s10995-023-03762-w (PMC10692244; doi:10.1007/s10995-023-03762-w)
Supplement: Supplementary file 1 — Supplementary file1 (PDF 126 kb) [file 10995_2023_3762_MOESM1_ESM.pdf]

Online Resource 1. Conceptual Framework for Implementation Research (CFIR) domains and corresponding example interview questions.

Figure caption: Three boxes each represent one CFIR domain: 1) Intervention; 2) Individuals Involved; and 3) Setting, which in this context refers to co-location of pediatric care with maternal OUD treatment. Arrows point to example interview questions relating to each domain.

Article title: Group well child care for mothers with opioid use disorder: Framework for implementation

Journal name: Maternal and Child Health Journal

Authors: Neera Goyal, MD, MSc, Meghan Gannon, PhD, Erica Sood, PhD, Grace Harris, Elizabeth Franko, Diane J. Abatemarco, PhD, MSW, Dennis J. Hand, PhD, Susan Leib MD, Vanessa L. Short, PhD, MPH

Corresponding author affiliation and contact: Nemours Children's Health, neera.goyal@nemours.org

		Example interview prompts
INTERVENTION 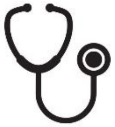	• Familiarity with intervention<br>• Perceived benefits of intervention for mothers in treatment for OUD and their children<br>• Potential negative outcomes of intervention<br>• Perceived difficulty/complexity of intervention	**Q1.** Compared with traditional care, how difficult does group well child care seem to you?<br>**Q2.** What might be some benefits of this approach for children and mothers in treatment for OUD?
INDIVIDUALS INVOLVED	• Interest and enthusiasm in the intervention<br>• Confidence and self-efficacy to participate<br>• Barriers to participate<br>• Personal benefit to participation	**Q1.** How interested would you personally be in conducting group well child care visits? Why or why not?<br>**Q2.** How confident do you feel that you could lead a group well child care visit for mothers with OUD and their children?
SETTING 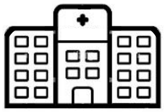	• Infrastructure to support intervention<br>• Collaboration between pediatric and maternal OUD programs	**Q1.** What resources would be needed for your practice to successfully provide group well child care?<br>**Q2.** How would you describe the current relationship between your pediatric office and the [maternal OUD] treatment program?
